# Color Modifications of a Maxillofacial Silicone Elastomer under the Effect of Cigarette Smoke

**DOI:** 10.3390/ma17164089

**Published:** 2024-08-17

**Authors:** Anca Irina Gradinariu, Alexandru-Constantin Stoica, Alexandra Bargan, Carmen Racles, Carmen Gabriela Stelea, Victor Vlad Costan

**Affiliations:** 1 Department of Oral and Maxillofacial Surgery, “Grigore T. Popa” University of Medicine and Pharmacy, 16 University Street, 700511 Iasi, Romania; anca-gradinariu@umfiasi.ro (A.I.G.); carmen.stelea@umfiasi.ro (C.G.S.); victor.costan@umfiasi.ro (V.V.C.); 2 Department of Inorganic Polymers, “Petru Poni” Institute of Macromolecular Chemistry, 41A Grigore Ghica Voda Alley, 700487 Iasi, Romania; anistor@icmpp.ro

**Keywords:** maxillofacial silicone, cigarette smoke, color modification, adsorption

## Abstract

Although it is known (from the observations of medical professionals) that cigarette smoke negatively affects maxillofacial prostheses, especially through staining/discoloration, systematic research in this regard is limited. Herein, the color modifications of M511 maxillofacial silicone, unpigmented and pigmented with red or skin tone pigments, covered with mattifiers, or with makeup and mattifiers, and directly exposed to cigarette smoke, were investigated by spectrophotometric measurements in the CIELab and RGB color systems. The changes in color parameters are comparatively discussed, showing that the base silicone material without pigmentation and coating undergoes the most significant modifications. Visible and clinically unacceptable changes occurred after direct exposure to only 20 cigarettes. By coating and application of makeup, the material is more resistant to color changes, which suggests that surface treatments provide increased protection to adsorption of the smoke components. The dynamic water vapor sorption (DVS) measurements indicate a decrease of the sorption capacity in pigmented versus unpigmented elastomers, in line with the changes in color parameters.

## 1. Introduction

As a result of increasing incidence of cancer, congenital defects, traumas, and accidents, certain defects on the head or neck regions, which cannot be treated surgically, are resolved with the use of maxillofacial prostheses. Since the 1960s, most external facial prostheses (ex vivo), or parts of them, have been made of silicone elastomers [1], which exhibit special, very useful properties [2,3], such as physiological inertness, excellent compatibility with blood, very low toxicity, anti-adhesive properties, and mechanical properties similar to the human tissues, and are adjustable within relatively large limits. In addition, silicones are lightweight, easy to manipulate and clean, and involve relatively simple manufacturing (including pigmentation) in the prosthesis handcrafting. Given the specificity of maxillofacial prostheses, they are very important in restoring the quality of life of affected patients, their role being both functional and aesthetic.

There are numerous brands of maxillofacial silicones, each with specific curing mechanisms and conditions and with a large diversity of additives, including pigments, which are essential in the desired aesthetics. Although acknowledged as the best choice in many situations, the silicone-based maxillofacial prostheses suffer from a relatively short life in service, roughly between 7 and 24 months [4], depending on several external factors, like UV radiation, extreme temperatures, dampness, biological fluids, soaps and disinfectants, air pollutants, and various mechanical stresses [5]. The main reasons for the necessity of replacement include color changes, poor maintenance (leading to the formation of biofilms), and mechanical deterioration.

The effect of natural weathering [6,7,8] or artificial aging [7,9,10,11,12] on the long-term use of silicone maxillofacial prostheses has been intensively investigated. The main studied aspect is the color change (discoloration) [6,7,8,9,10,12], which is incriminated based on patients’ feedback, while other reports focus on deterioration of the mechanical properties [6,9,12,13,14,15]. The color changes after exposure to different factors (weathering, disinfecting solutions, etc.) are assessed using spectrophotometric methods. One of these methods, CIE Lab, which is largely used, numerically describes the color variation between samples, expressed by the parameter ΔE [7]. It has been established that, from a clinical point of view, values higher than 3 for this parameter are not acceptable [10]. It is known that ceramic pigments lead to good color stability of materials, while pigments used in make-up or intrinsic organic pigments were found to be much more prone to degradation, thus to color changes [1,10]. Studies report that the use of additives such as opacifiers, TiO_2_ nanoparticles, and BaTiO_3_ contributes to color stabilization [1,12]. However, since unpigmented specimens experienced color change as well [7], the degradation of the silicone material itself seems to be the most important issue. It was established that photo-oxidation is the main degradative process in natural or artificial aging [14,16], while the instability in acidic medium affects the silicones when exposed to simulated body fluids or acidic solutions [15,17].

Very little is known about the influence that the cigarette smoke may have on silicone elastomers. In the meantime, smoking is acknowledged in clinical observations as a factor producing staining and discoloration to maxillofacial prostheses [7,18] and contributing to their accentuated and accelerated deterioration. The cigarette smoke is a very complex mixture of compounds, some of them extremely toxic [19,20]. Therefore, besides the unpleasant discoloration of the prostheses, the adsorption and persistence of such compounds within the silicone maxillofacial prosthesis material should be a concern, as well as the alteration of other properties. To date, the role of cigarette smoke in deterioration of silicone-based maxillofacial prostheses has not been systematically studied. The color stability of Silastic 44210 under exposure to cigarette smoke was first investigated in 1983 by Yu et al. [21]. The spectrophotometric analyses revealed a slight increase of the dominant wavelength and a large increase in color saturation, based on the CIE1931 color system. Since then, no other studies were reported till 2022, when Ozyemisci and Kurt [22] exposed the maxillofacial silicone M511 to the cigarette smoke and evaluated the color changes and the efficiency of hand soap and chlorhexidine gluconate mouthwash in removing the stains. The authors reported significant modifications, over the clinically acceptable threshold, in all the samples, based on CIE Lab (ΔE) and CIEDE2000 (ΔE00) color measurement formulas.

Our recent research [23] showed more complex implications of the cigarette smoke on maxillofacial silicone M511. For example, the roughness increased, the local mechanical properties changed, and the contact angle decreased after exposure to the smoke. Structural changes in the silicone material, like secondary cross-linking, were supposed to be based on FTIR and thermal analyses. The persistence of lead traces was evidenced in the sample exposed to cigarette smoke after extraction with solvents, and several adsorbed organic compounds were identified.

Herein, we extend our investigation on the color modification of M511 maxillofacial silicone, unpigmented and pigmented with red or skin tone pigments, covered with mattifiers, or with makeup and mattifiers. The spectrophotometric measurements in the CIELab and RGB color systems are reported, and the changes in color parameters are comparatively discussed. Significant effects of the cigarette smoke on the base material with color modifications over the clinically acceptable threshold were registered, but these diminished after pigmentation, coating, or makeup with coating. The reduction of the sorption capacity after pigmentation is considered responsible for increased resistance of the base elastomer to smoke staining.

## 2. Materials and Methods

The raw materials were purchased from Technovent (Factor II): maxillofacial silicone elastomer M511 (two components), intrinsic pigments FI-200, FI-204, and FI-SK13, silicone dispersion TS-564 for surface treatment, matting dispersion MD-564, and medical silicone adhesive A-564.

### 2.1. Samples Preparation and Exposure to the Smoke

**Scheme 1:**
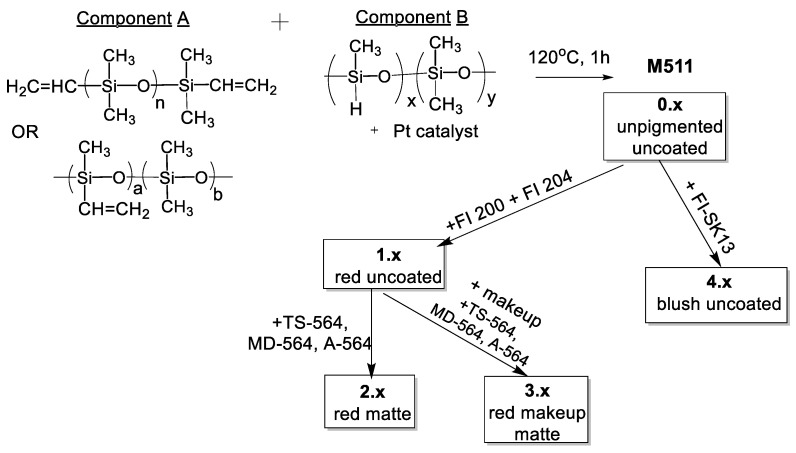
Schematic representation of the addition cross-linking, the presumptive structures of the main silicone components in the maxillofacial elastomer, and sample coding.

The two components of the base silicone were manually mixed in 10:1 proportion by weight of component A to component B. The codes and pigmentation are depicted in Scheme 1. For the “0” series, no pigment was added, while for the other specimens, intrinsic pigments were added and manually mixed to homogeneous color, that is, white pigment FI-200 and red pigment FI-204 for the “red” samples and FI-SK13 pigment for the “blush” samples, respectively. The mixtures were poured into small Teflon molds, leveled with a spatula, and vulcanized at 120 °C for 1 h in a normal oven without applying pressure or vacuum. The sample size was 10 mm × 5 mm (diameter × thickness). For comparison, a film of ca. 400 μm in thickness was also prepared from the unpigmented material. After curing, part of the pigmented samples were kept as such, without surface treatment (series “1” and “4”), and others were treated at the surface, as follows. For series “2”, a layer of acetoxy silicone dispersion TS-564 was applied by brushing and left in a normal atmosphere to cure for 1 h. Then a thin layer of the two component mattifier (consisting of silicone matting dispersion MD-564 and medical silicone adhesive A-564 in a 5:1 wt ratio) was applied by brushing. The material was left in ambient conditions till the next day to ensure the curing of the surface layer. For series “3”, a makeup (blush) was applied, prior to surface coating as described for series “2”.

From each composition, that is, unpigmented uncoated (“0.x”), red uncoated (“1.x”), red matte (“2.x”), red makeup matte (“3.x”), and blush uncoated (“4.x”), one specimen was kept as control and others were exposed to the smoke of “x” cigarettes, as follows.

A miniature smoking chamber was manufactured, adapted from literature [23,24]. The system consisted of a 20 mL glass jar with two rubber insets in the lid, a trap, and a water pump. The samples were placed individually inside the smoking chamber and were exposed to 10 or 20 cigarettes, which were burned one by one during 7–8 min. Other samples (0.60 thin film and 4.60) were placed inside the trap during the burn of 60 cigarettes. The ensemble was placed outdoors on a private property. After exposure to smoke, the samples were analyzed without further treatment aside from a brief rinse with distilled water.

### 2.2. Color Measurements

The measurements were carried out with a Specord 210 Plus spectrophotometer equipped with the integrating sphere in the absorbance module. The L, a, b parameters were calculated with the colorimetry function in the WinAspect Plus 4.0 software, using an angle of observer = 2° and standard illuminant A.

The overall color change (ΔE, L a b) and lightfastness (ΔE*, a b) of unpigmented and pigmented samples after exposure to the smoke were calculated with Equations (1) and (2), taking as reference the corresponding un-exposed sample.

ΔE = [(ΔL)^2^ + (Δa)^2^ + (Δb)^2^]^1/2^ (1)

ΔE* = [(Δa)^2^ + (Δb)^2^]^1/2^ (2)

where L, a, b are the CIELab coordinates: L defines lightness (+L) and darkness (–L); a defines the red (+a) and green (–a) chromatic component; b defines the yellow (+b) and blue (–b) chromatic component of the CIE color system.

ΔL = L − L_ref_; Δa = a − a_ref_; Δb = b − b_ref_ (3)

### 2.3. DVS Analysis

Dynamic water vapor sorption measurements were done using a fully automated compact bench-top DVS analyzer, IGASorp from Hiden Analytical (Warrington, UK). As the humidity is varied in the sample chamber at a constant temperature, the weight change is measured with the incorporated ultrasensitive microbalance. The first step of the measurement consisted of drying the samples at 25 °C in nitrogen flow (250 mL/min) until the weight was in equilibrium at a relative humidity (RH) smaller than 1%. Next, the adsorption isotherm was registered by gradually increasing the RH from 0 to 90% in 10% humidity steps with a pre-established equilibrium time between 20 and 40 min, when the sorption equilibrium was obtained for each RH value. The RH was then decreased similarly, and the desorption curves were registered [25].

## 3. Results and Discussion

The maxillofacial silicone elastomer samples were prepared by mixing the two components, with or without pigments, followed by high temperature vulcanization by addition (hydrosilylation) reaction, as exemplified in Scheme 1. The structure of the main components is not precisely known, but based on NMR analyses, we identified ca. 0.6 mol% vinyl groups (which could be attached in telechelic or pendant position) in Component A and ca. 14 mol% Si-H groups in Component B. Other additives could be present (especially silica), but those are not involved in the cross-linking reaction. After curing, part of the samples was coated using mattifiers, or makeup was applied prior to matiffiers, as depicted in Scheme 1. The silicone pieces were exposed to the smoke from “x” cigarettes, then analyzed spectrophotometrically.

We previously investigated similar samples with a wide set of methods in order to understand the complex effect that cigarette smoke might have on the maxillofacial silicone material [23]. The AFM analyses revealed surface changes in terms of roughness and local mechanical properties. The water contact angle decreased in most cases but remained practically unchanged in the mattified samples, indicating weaker adhesion of the hydrophilic or hygroscopic compounds [26]. The hardness slightly decreased after exposure to the smoke. The FTIR, TGA-DTG, and DSC data indicated structural changes within the base material, which were assigned to secondary cross-linking. Such chemical reactions could be possible by two mechanisms: (a) the Si-H groups that remained unreacted might undergo self-cross-linking by dehydrocoupling reactions in the presence of atmospheric moisture due to the decrease of the silicone hydrophobic character; (b) the methyl groups might be involved in radical cross-linking since free radicals were reported to form during cigarette burning [27], and this process might be prompted by exposure to sunlight and increased local temperature during the accelerated smoking experiment. In addition, traces of lead were detected by XRF in the sample exposed to cigarette smoke after being extracted with solvents. The organic solvent extracts were submitted to ESI-MS, MALDI-MS, and NMR spectral analyses. Besides nicotine, its metabolites, and other organic compounds from the cigarette smoke, we identified large amounts of siloxane compounds [5,23], which indicate that the base silicones contain non-cross-linkable materials, which could be expelled in time, altering the properties of the prostheses.

The unpigmented material was also processed as a film and analyzed in transmittance mode for optical and chromatic changes [23] based on CIE 1931 xyz chromaticity and CIE 1976 UCS (u′, v′) chromaticity diagrams before and after being exposed to 60 cigarettes. Important changes were observed: the dominant wavelength increased from 494 nm in the pristine sample up to 564 nm, and the illuminance value decreased, indicating approximately 23% loss of transparency. The color temperature for the 0.60 sample was significantly lower than for the 0.0 control film. This method was only suitable for transparent samples due to technical limitations; thus, the pigmented and coated samples could not be measured. Herein the CIE Lab color system was used, and the measured parameters are presented in Table 1 for all the samples. The overall color change (ΔE, L a b) and lightfastness (ΔE*, a b) were calculated by reporting the registered values to the appropriate control sample in each series.

Our results showed significant color and lightness changes in the unpigmented silicone, both as thin and thick samples. The lightness decreased (ΔL negative) and the yellow (Δb) and red (Δa) chromatic components increased after exposure to the smoke. The ΔE values for the unpigmented samples (Table 1) are very high, clinically unacceptable, irrespective of the formula used for calculation (with or without considering lightness). This is in line with the report of Ozyemisci and Kurt [22], where a very high ΔE value of 46 was found for the control sample without pigmentation, after exposure to 200 cigarettes. The modifications of the color parameters for the silicone film (0.60) are consistent with the previous observations made in transmitance mode [23], where significant loss in transparency and modification of the color towards yellow were found. The ΔE value obtained herein from direct measurements in absorbance mode is higher than resulted previously from the calculations, due to different methods of measurement.

In the pigmented uncoated samples (series “1” and “4”), the color differences had lower values compared to the neat silicone, and increased with the number of cigarettes (Table 1). However, all the ΔE values were higher than clinically acceptable, irrespective of the pigments used. The lightness decreased, and the red and yellow tints accentuated.

When comparing the two samples exposed to 60 cigarettes (both being uncoated), it can be observed that the unpigmented silicone was more affected by the smoke than the blush pigmented one. Leaving aside the difference in thickness between samples 0.60 and 4.60, the tendency remains when samples with equal thickness are compared, since 4.60 exhibited the same ΔE value as the unpigmented 0.20, showing that 60 cigarettes were necessary for the pigmented sample to reach the same level of modification as the unpigmented one exposed to only 20 cigarettes. The same tendency and even larger difference between pigmented and unpigmented control samples was reported previously [22] after 200 cigarettes. The pigments, especially the organic ones, are considered less resistant to UV radiation [18], but when it comes to cigarette smoke, the pigmented samples were obviously less affected. So, the main reason for the poor resistance to this pollutant is the base silicone material, since it exhibited the largest color modification.

It is known that PDMS is very permeable to diffusion of different substances, including gases, water vapors, and drugs [28]. For example, its permeability to oxygen is 25-fold higher than that of natural rubber, and despite the pronounced hydrophobic character of the silicone rubber, its water vapor permeability is very high, comparable to collagen and three-fold higher than that of polystyrene [29]. This behavior can explain the accumulation of soluble and volatile compounds within the material upon exposure to the cigarette smoke. The pores that form during crosslinking are also a favorable factor for adsorption of various compounds.

**Figure 1:**
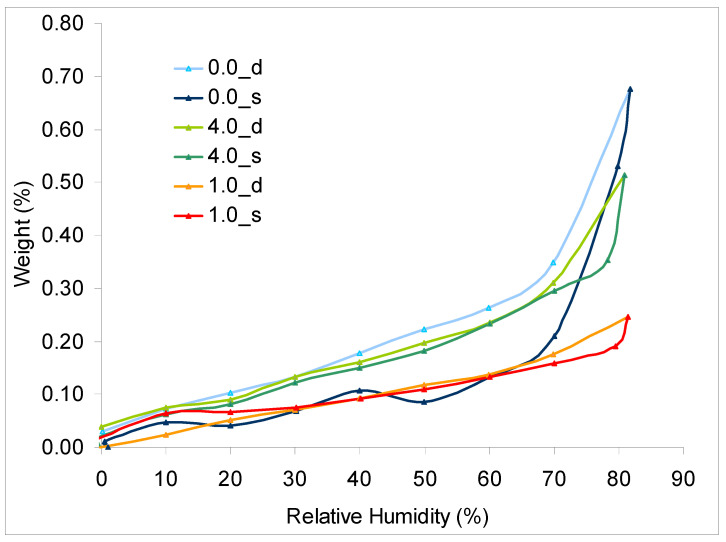
Dynamic vapor sorption isotherms of uncoated samples (“s” stands for sorption and “d” for desorption branch).

Dynamic vapor sorption (DVS) measurements gave some information on the sorption capacity in bulk of the uncoated, unpigmented, and pigmented materials before being exposed to the smoke. The experimental results are depicted in Figure 1, as the sorption-desorption isotherms. The water vapor sorption capacity in the dynamic regime was below 0.7% for all the samples and decreased in the order: 0.0 > 4.0 > 1.0. This is in line with the trend of color modification after exposure to the smoke (Table 1), which decreased in the pigmented samples.

The low water vapor sorption capacity and the shape of the sorption-desorption isotherms, which are assigned to type V isotherms according to the IUPAC classification [30], show the hydrophobic character of the silicone samples and also suggest the presence of pores. The hysteresis loop is smaller for samples 4.0 and 1.0 than for sample 0.0, which is consequence of a decreased porosity.

**Figure 2:**
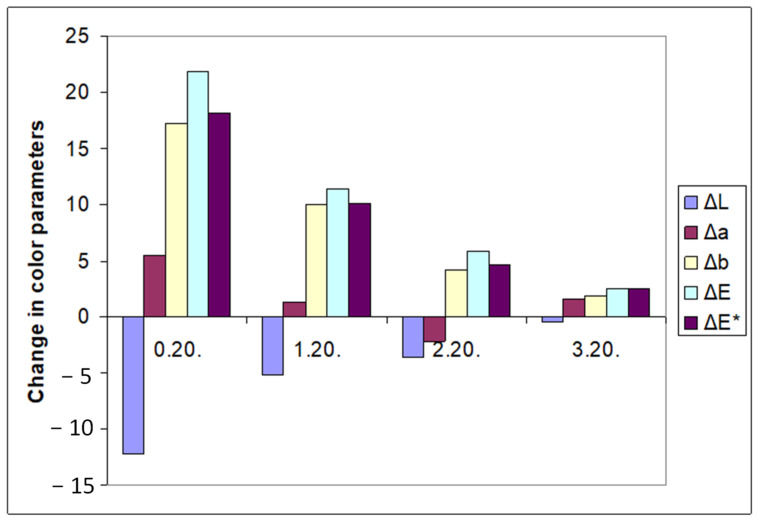
The modification of different color parameters of unpigmented and red samples after being exposed to the smoke of 20 cigarettes.

The effect of 20 cigarettes on the color changes of differently treated samples is compared in Figure 2. The graph very clearly shows the gradual decrease in color parameter modifications (ΔL, ΔE, and ΔE*), from unpigmented uncoated, red pigmented, to coated, and makeup coated samples. Thus, the effect of the intrinsic pigment, matiffier, and extrinsic pigment plus mattifier was to progressively enhance the resistance to the discoloration under the action of the smoke. The pigments partially fill the voids in the silicone material and reduce the water sorption capacity, as suggested by DVS analysis, while the mattifiers seal the material and modify the surface properties, as mentioned before. Since adsorption of the volatile and water soluble compounds is probably the main physical phenomenon that changes the appearance of the materials submitted to cigarette smoke, the overall effect of pigments and coatings seems to be the reduction of the adsorption capacity of the material, thus offering protection against the components of the smoke.

The makeup layer applied underneath the mattifier had a significant positive effect on our samples, ensuring clinically acceptable modifications, with ΔE < 3 after exposure to 20 cigarettes, contrary to all the other samples, which exhibited clinically unacceptable modifications (Table 1). According to ΔE* values, the coated samples felt into ASTM category II (very good lightfastness), having ΔE* between 4 and 8, and even into category I (excellent lightfastness, with ΔE* < 4) [7] in the case of sample 3.20, with makeup and mattifier. The uncoated samples, on the other side, showed high to very high changes in lightfastness, corresponding to categories III or IV. Thus we can conclude that each step of the handcrafting process of maxillofacial prostheses (i.e., pigmentation, coating with mattifier, and applying makeup before mattifier) can progressively improve the color stability of the silicone base material when exposed to the cigarette smoke.

The mattifiers are in fact coatings, forming a pellicle that protects the bulk material. The makeup, besides aesthetics, has the role of blocking the pores on the surface. Both the mattifiers and the makeup limit the penetration of the volatiles and tar, which can stain the silicone. These surface treatments are mandatory and are used as routine in practice. It seems that they form a protective layer against the cigarette smoke and possibly other factors. Similarly, it was previously observed that “extrinsic coloration may reduce the incidence of discoloration in maxillofacial prosthesis” [31].

**Figure 3:**
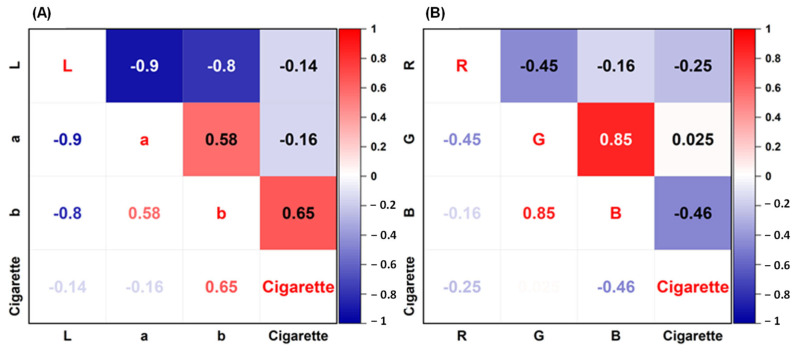
The Pearson correlation matrix for: (**A**) the CIELab system and (**B**) RGB system.

Due to the limited number of samples investigated, a statistical analysis of the obtained data is not possible and should be the object of more thorough, dedicated studies. In order to get an overview of the effect that the cigarette smoke had on the studied materials, the color data were examined through the Pearson correlation matrix (coefficient). This test measures the degree and direction of the relationship between two variables and is used to determine whether there is a statistical association between them. Pearson correlation values can fluctuate between −1 and 1, with 0 representing zero correlation [32]. For the CIELab system (Figure 3A), the correlations were weak negative between the number of cigarettes and the parameters L and a, and medium-strong positive between the number of cigarettes and the parameter b. The weak negative correlation between the number of cigarettes and L suggests that with exposure of materials to cigarette smoke, they become darker or lose their original luster. The weak negative correlation with parameter a suggests that the material loses the contribution of the red component in the color. The medium-strong positive correlation with the b parameter suggests that the material becomes much yellower.

The measured RGB color parameters are presented in Table 2, together with the resulted colors. The measured colors are very similar to the true colors observed visually. The Pearson test was also applied for the RGB system (Figure 3B) and revealed a medium negative correlation between the number of cigarettes and the B parameter (specific to the pure blue color), a weak negative correlation with the R parameter (specific to the pure red color), and no correlation with the G parameter (specific to the pure green color). These correlations suggest that upon the action of cigarette smoke on the studied materials, they keep constant their percentage of green and decrease their percentage of red and especially blue, in practice translating into a change in color towards brown.

Based on the data presented herein and in a more detailed study [23], we consider that the discoloration under the influence of cigarette smoke is mainly a physical process, consisting in the adsorption of different compounds and materials (including tar, for example), which stain the surface but also penetrate in the bulk, especially when a protective coating (like the mattifier) is missing. This hypothesis is sustained by the extraction of a complex mixture of soluble compounds in THF [23] and by the previous studies [21,22], which proposed different methods for removing the stains. Although such methods may be questionable because they may as well remove unbonded additives and deteriorate other properties [5], their relative success in restoring the initial color of the prosthesis proves that the discoloration was unlikely to be based on chemical reactions.

## 4. Conclusions

The maxillofacial silicone M511 unpigmented, pigmented, uncoated, and coated was evaluated for color changes after being exposed to cigarette smoke. The measurements showed very high changes in color parameters for the base material without pigments and coatings and decreasing, yet over acceptable, values of ΔL, ΔE, and ΔE* when the base was pigmented or coated. Only when makeup was applied underneath the mattifier layer, the samples exhibited clinically acceptable changes after being exposed to the smoke of 20 cigarettes. The DVS analysis showed a decrease in water vapor sorption capacity in the pigmented materials compared with the unpigmented base. The data suggest that, by diminishing the adsorption capacity, the material is more resistant to the smoke staining. This study brings new insights into the effect of the cigarette smoke on a medical silicone elastomer, an effect that is scarcely investigated, but still an important issue concerning the long term stability of maxillofacial prostheses.

## Figures and Tables

**Table 1:** The color parameters of the samples before and after exposure to the cigarette smoke.

Code	L	a	b	ΔL	Δa	Δb	ΔE	ΔE*
unpigmented								
0.10	96.54	1.01	7.04					
0.20	84.31	6.48	24.29	−12.22	5.47	17.26	21.85	18.11
0.0 film	97.67	−0.47	3.29					
0.60 film	89.50	9.37	33.63	−8.16	9.85	30.34	32.93	31.90
red								
1.0	79.79	50.52	22.83					
1.10	75.62	53.25	29.73	−4.17	2.72	6.90	8.51	7.42
1.20	74.63	51.80	32.87	−5.16	1.28	10.04	11.36	10.12
red matte								
2.0	78.84	52.90	25.66					
2.20	75.31	50.69	29.81	−3.54	−2.20	4.16	5.89	4.70
red makeup matte								
3.0	77.76	40.05	24.01					
3.20	77.31	41.60	25.89	−0.46	1.54	1.88	2.474	2.43
blush								
4.0	92.05	8.24	16.48					
4.60	81.74	18.09	32.18	−10.31	9.85	15.70	21.21	18.53

**Table 2:** The RGB color parameters, and the resulted colors (pasted as background in the second column).

Code	Color	R	G	B
unpigmented				
0.0 (film)		248	249	242
0.60 (film)		251	217	159
0.10		249	244	231
0.20		229	205	164
red				
1.0		255	146	162
1.10		255	131	139
1.20		255	130	130
red matte				
2.0		255	140	155
2.20		255	134	137
red makeup matte				
3.0		255	154	152
3.20		255	151	148
blush				
4.0		253	226	200
4.60		244	188	143

## Data Availability

The original contributions presented in the study are included in the article, further inquiries can be directed to the corresponding authors.
